# Efficacy of recombinant bovine basic fibroblast growth factor to reduce hemorrhage after cervical loop electrosurgical excision procedure

**DOI:** 10.1186/s12905-023-02474-1

**Published:** 2023-07-21

**Authors:** Chongrong Weng, Jian Xu, Hua Yang, Panxia Deng, Honghui Ou, Yue Xu, Yuan Zhuang, Huilong Nie

**Affiliations:** Department of Gynecology, The Fifth Affiliated Hospital of Sun Yat-sen University, Zhuhai, 519000 People’s Republic of China

**Keywords:** Recombinant bovine basic fibroblast growth factor (rbFGF), Cervical loop electrosurgical excision procedure (LEEP), Hemorrhage, Wound healing, Hemostasis

## Abstract

**Objective:**

It has been reported that recombinant bovine basic fibroblast growth factor (rbFGF) may possess possible biological functions in promoting the process of wound healing. Consequently, our study aimed to investigate the hemostatic effect of topically applied rbFGF in patients who underwent a loop electrosurgical excision procedure (LEEP).

**Methods:**

In this retrospective analysis, we meticulously examined clinicopathologic data from a cohort of 90 patients who underwent LEEP at our institution between 2020 and 2021. Subsequently, we conducted inquiries with the patients to ascertain the degree of vaginal bleeding experienced during the postoperative periods of 3 and 6 weeks, comparing it to their preoperative menstrual flow. The magnitude of the menstrual volume alteration was then quantified using a menstrual volume multiplier(MVM). The primary endpoints of our investigation were to assess the hemostatic effect of rbFGF by means of evaluating the MVM. Additionally, the secondary endpoints encompassed the assessment of treatment-related side effects of such as infection and dysmenorrhea.

**Results:**

Our findings demonstrated a significant reduction in hemorrhage following cervical LEEP. Specifically, in the per-protocol analysis, the study group exhibited a statistically significantly decrease in MVM after 3 weeks (0 [0–0] vs. 1 [0–1], respectively; *p* < 0.001) and after 6 weeks (1 [1] vs. 2 [1–3], respectively; *p* < 0.001) of the procedure. No notable disparities were observed in the remaining outcomes between the two groups. Moreover, a logistic regression analysis was employed to explore the relationship between significant bleeding and rbFGF treatment (*p* < 0.001, OR = -2.47, 95% CI -4.07 ~-1.21), while controlling for confounding factors such as age, BMI, and surgical specimen.

**Conclusions:**

In conclusion, our study findings highlight that the application of recombinant bovine basic fibroblast growth factorcan effectively mitigate hemorrhage subsequent to cervical loop electrosurgical excision procedure.

## Introduction

Cervical intraepithelial neoplasia (CIN) represents a premalignant lesion diagnosed through histological examination. Treatment options for CIN include cryotherapy, loop electrosurgical excision procedure (LEEP), and cold knife conization (CKC) [1]. Since 1989, LEEP has emerged as a frequently employed management modality for women with cervical intraepithelial neoplasia [2]. When compared with other types of conization therapy, LEEP offers the advantages of cost-effectiveness, expeditiousness, simplicity, and the procurement of high-quality specimens [3, 4]. Additionally, LEEP is associated with l ower recurrence rates of CIN 2–3 and reduced incidences of both major and minor complications [5]. Y Hurtado-Roca, et al. conducted a comprehensive review of 72 studies spanning the period from January 1993 to September 2018 (covering the past 25 years), concluding that the utilization of LEEP for managing premalignant lesions significantly reduces the risk of infections (*p* ≤ 0.001) [6]. However, the review also highlights that LEEP is linked to an increased risk of minor bleeding [6]. In clinical practice, we have observed that delayed hemorrhage following LEEP engenders patiens anxiety and disrupts daily activities.

Several studies have investigated interventions aimed at mitigating blood loss during the treatment of cervical intraepithelial neoplasia, with a primary focus on knife and laser cone biopsy. These investigations have revealed that vasopressin, tranexamic acid, hemostatic sutures, Amino-Cerv, Monsel’s solution, Tissucol-R [7] exhibit certain degrees of efficacy in reducing vaginal bleeding [8]. Nonetheless, only a limited number of studieshave specifically addressed the prevention of postoperative hemorrhage after LEEP, demonstrating that Tachosil (Nycomed, Zurich, Switzerland) [9, 10] and chitosan tampon (Hemoblock-Tampon; Incore, Daegu, Korea) [11] effectively mitigate delayed vaginal bleeding.

Fibroblast growth factor is a class of significant growth factors involved in the wound repair process in organisms [12]. Topical application of recombinant bovine basic fibroblast growth factor (rbFGF) has gained widespread use in the management of burns, fresh wounds, and chronic wounds [12]. We posit that the topical application of rbFGF on cervical wounds facilitates wound repair and prevents delayed vaginal bleeding. However, to our knowledge, no studies have addressed this issue. Therefore, we conducted a retrospective study to elucidate the efficacy of rbFGF in reducing hemorrhage subsequent to LEEP.

## Materials and methods

### RbFGF gel

The API of Beifuxin Gel (Zhuhai Essex Bio-Pharmaceutical Co., Ltd., Zhuhai, China) is recombinant bovine basic fibroblast growth factor (rbFGF), which can stimulate the repair and regeneration of tissues and cells derived from the mesoderm and ectoderm (such as epithelial cells, dermal cells, fibroblasts, vascular endothelial cells, osteoblasts, nerve cells, etc.). The specific combination of rbFGF with the wounded target cell surface can trigger the cells to repair actively and stimulate cell division, proliferation, migration, and differentiation, accelerating wound healing and improving the healing quality.

### Design of the study

This study retrospectively collected information from 90 patients who underwent loop electrosurgical excision procedure (LEEP) for cervical conization at our medical institution between 2020 and 2021. The inclusion criteria consisted of patients who met all the indications for cervical conization and underwent LEEP surgery at our institution. Additionally, these patients had follow-up data available at 3 and 6 weeks postoperatively, and were premenopausal. The exclusion criteria included patients with comorbidities such as coagulation disorders, patients with a history of medication usage that could impact bleeding or other long-term medications, and other factors that could potentially affect data collection.

All patients underwent LEEP surgery using an electrosurgical knife for the procedure and achieved hemostasis through electrocoagulation. Vaginal packing was subsequently applied.

**Fig. 1:**
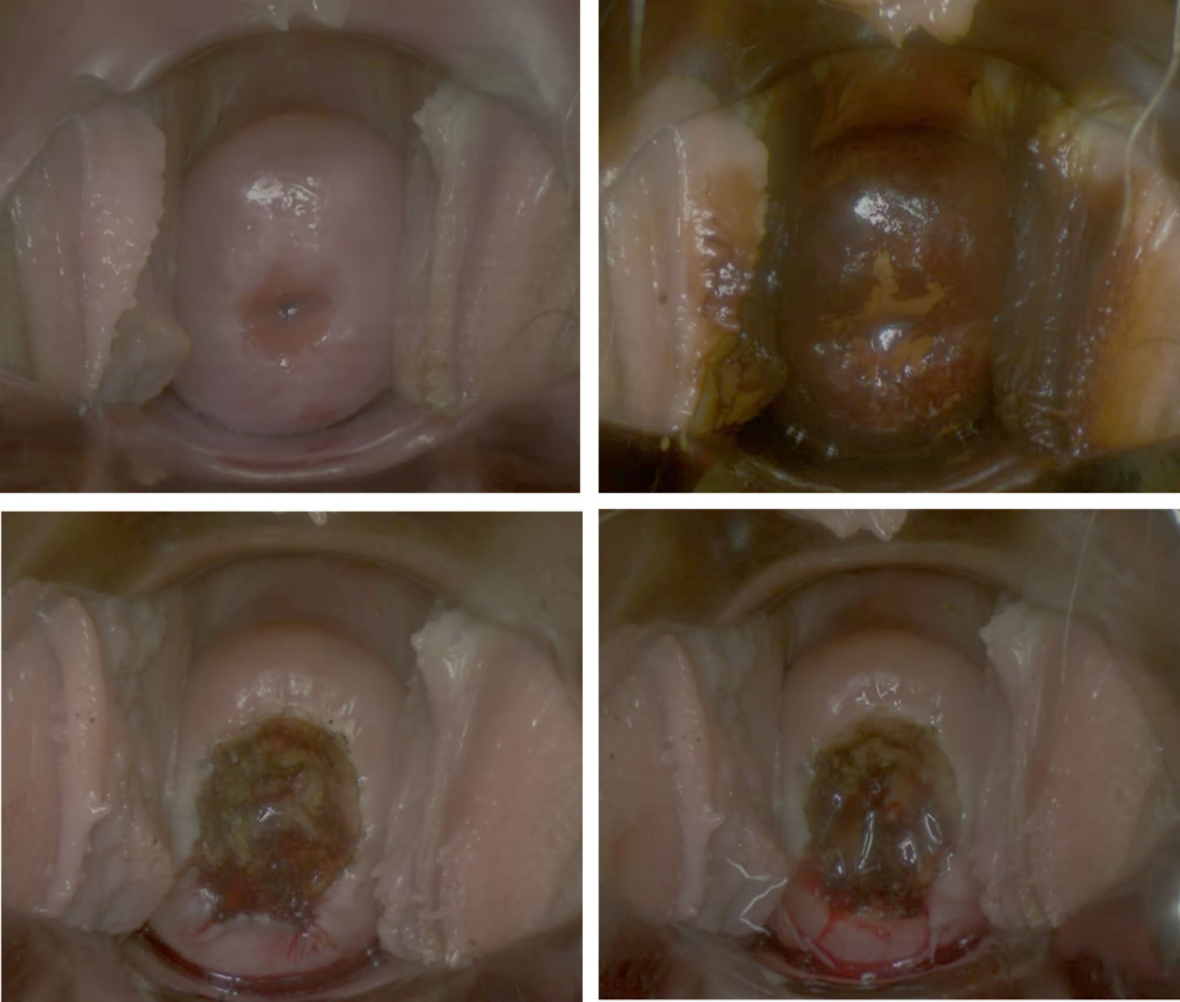
LEEP procedure and rbFGF gel application

In the experimental group, a subset of patients received local application of recombinant human basic fibroblast growth factor (rbFGF) on the cervix after the use of the electrosurgical knife and before the placement of vaginal packing (Fig. 1).

Postoperative follow-up involved telephone interviews conducted at 3 and 6 weeks after surgery. Nurses inquired about postoperative bleeding, infection, and other relevant conditions to assess the need for further intervention. When assessing postoperative bleeding, nurses asked patients about the volume of bleeding in relation to their normal menstrual flow before the procedure. This assessment involved calculating a value called menstrual volume multiple (MVM), which represents the ratio of postoperative vaginal bleeding volume to the patient’s normal menstrual flow. An MVM of 1 indicated that the postoperative vaginal bleeding volume was equivalent to the patient’s normal menstrual flow, while an MVM of 2 indicated that the postoperative vaginal bleeding volume was twice that of the patient’s normal menstrual flow. The MVM provided a straightforward and intuitive approach to quantifying postoperative vaginal bleeding, facilitating timely intervention when necessary.

### Statistical analysis

The statistical analyses entailed utilization of the Mann-Whitney U test to compare continuous variables that did not satisfy the Shapiro-Wilk normality test. For categorical data, the chi-square test or Fisher’s exact test (in cases of small counts) was employed to compare frequencies. To assess significant hemorrhage at the 6-week mark, a logistic regression model was employed, and the resulting estimated odds ratios (ORs) with corresponding 95% confidence intervals (95% CIs) were reported. All *p* values are 2-tailed and a *p* value < 0.05 denoted statistical significance. The statistical analyses were performed using the R software (version 4.2.0) and GraphPad Prism 7 software (version 7.0).

## Results

Ninety female participants were enrolled in this study, with a mean age of 37 years (range 21–49 years). All participants were premenopausal. Table 1 displays the patients’ characteristics and pathological features for both study groups. No significant differences were observed between the two groups in terms of- age, BMI, parity and histopathology. The patient population exhibited a well-balanced distribution.

**Table 1:** Baseline characteristics of study participants

	LEEP	LEEP with rbFGF	
Variables	control(n = 43)	treatment(n = 47)	*p*-value
Age(years)	37.0(32.0–40.0)	38.0(31.0–44.0)	0.5576
BMI(kg/m 2)	21.4(20.5–24.2)	21.8(20.7–23.0)	0.5771
Parity	1(1–1)	1(1–1)	0.9354
Histopathology			
≤LSIL	17(39.5)	16(34.0)	0.7481
≥HSIL	26(60.5)	31(66.0)	

LEEP, loop electrosurgical excision procedure

LSIL, low-grade squamous intraepithelial neoplasia

HSIL, high-grade squamous intraepithelial neoplasia

Data are presented as number (percentage) or median (interquartile range)

We did the LEEP procedure between two groups of patients. And both of them share nearly the same surgery characteristics, including operative time, intraoperative blood loss, diameter and depth of cervical cone specimens, the percentage of transformation zone visible, and positive margin (Table 2).

**Table 2:** The LEEP procedure between two group

	LEEP	LEEP with rbFGF	
Variables	control(n = 43)	treatment(n = 47)	*p*-value
Operative time(min)	20.0(16.0–30.0)	22.0(12.0–30.0)	0.7477
Intraoperative blood loss			
sponge weight(g)	10.0(10.0–20.0)	10.0(5.0–25.0)	0.9438
Surgical specimen			
Diameter(cm)	3.5(3.0-3.8)	3.5(2.0-3.5)	0.5555
Depth(cm)	1.5(1.25–1.5)	1.5(1.0-1.5)	0.7999
Transformation			
zone visible	38(88.4)	40(85.1)	0.8848
Positive margin	2(4.7)	1(2.1)	0.6043

LEEP, loop electrosurgical excision procedure

Data are presented as number (percentage) or median (interquartile range)

**Fig. 2:**
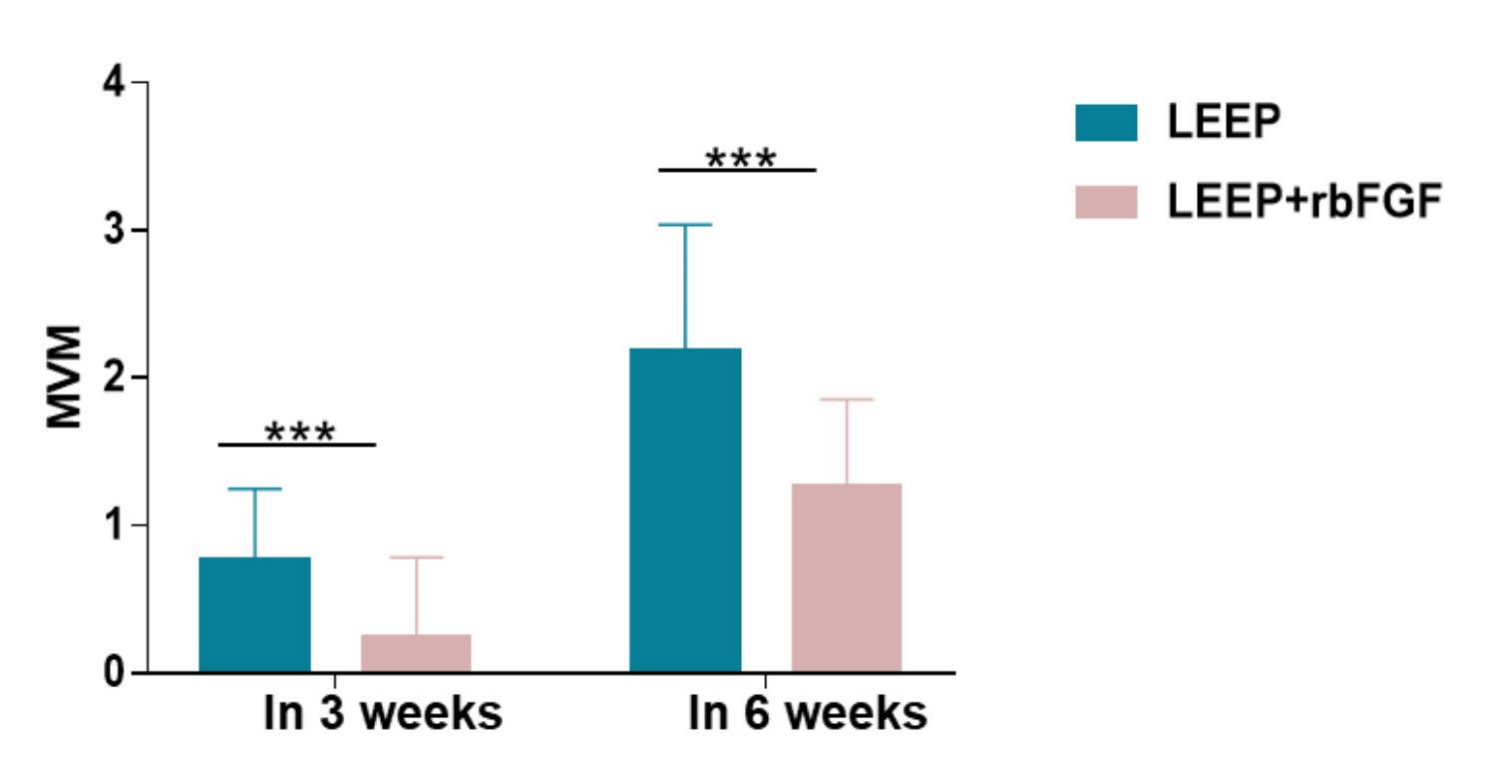
MVM in 3 weeks and 6 weeks between two groups. MVM, menstrual volume multiplier, the vaginal bleeding volume in 3 and 6 weeks postoperatively compared to menstruation before surgery. ***, *p*<0.001

Table 3 presents the outcome results of the study, including the potential side effects of the rbFGF treatment, namely dysmenorrhea (8 cases [18.6%] in the LEEP control group vs. 8 cases [17.0%] in the LEEP with rbFGF treatment group; *p* = 1) and postoperative infection (1 case [2.3%] in the LEEP control group vs. 0 cases [0.0%] in the LEEP with rbFGF treatment group; *p* = 0.478). the primary outcomes, postoperative hemorrhage, were also examined. theThe study group (using rbFGF after LEEP) exhibited a significant reduction in postoperative hemorrhage in 3 weeks (1 case [0–1] in the LEEP control group vs. 0 cases [0–0] in the LEEP with rbFGF treatment group; *p* < 0.01) (Fig. 2). Similarly, postoperative hemorrhage at 6 weeks decreased in patients receiving rbFGF treatment (2 cases [1–3] in the LEEP control group vs. 1 case [1] in the LEEP with rbFGF treatment group; *p* < 0.01) (Fig. 2). Patients were further stratified based on their reported MVM (menstrual volume multiplier) at 6 weeks, with MVM values greater than 2 indicating significant bleeding after surgery. The case group exhibited a substantially lower rate of patients with significant bleeding ((18 cases [41.9%] in the LEEP control group vs. 3 cases [6.4%] in the LEEP with rbFGF treatment group; *p* < 0.01)

**Table 3:** Outcome results of the study group

	LEEP	LEEP with rbFGF	
Variables	control(n = 43)	treatment(n = 47)	*p*-value
Dysmenorrhea	8(18.6)	8(17.0)	1
Postoperative infection	1(2.3)	0(0.0)	0.4778
Postoperative hemorrhage			
at 3 weeks	1(0–1)	0(0–0)	<0.001
at 6 weeks	2(1–3)	1(1–1)	<0.001
Significant bleeding	18(41.9)	3(6.4)	<0.001

LEEP, loop electrosurgical excision procedure

Data are presented as number (percentage) or median (interquartile range)

**Fig. 3:**
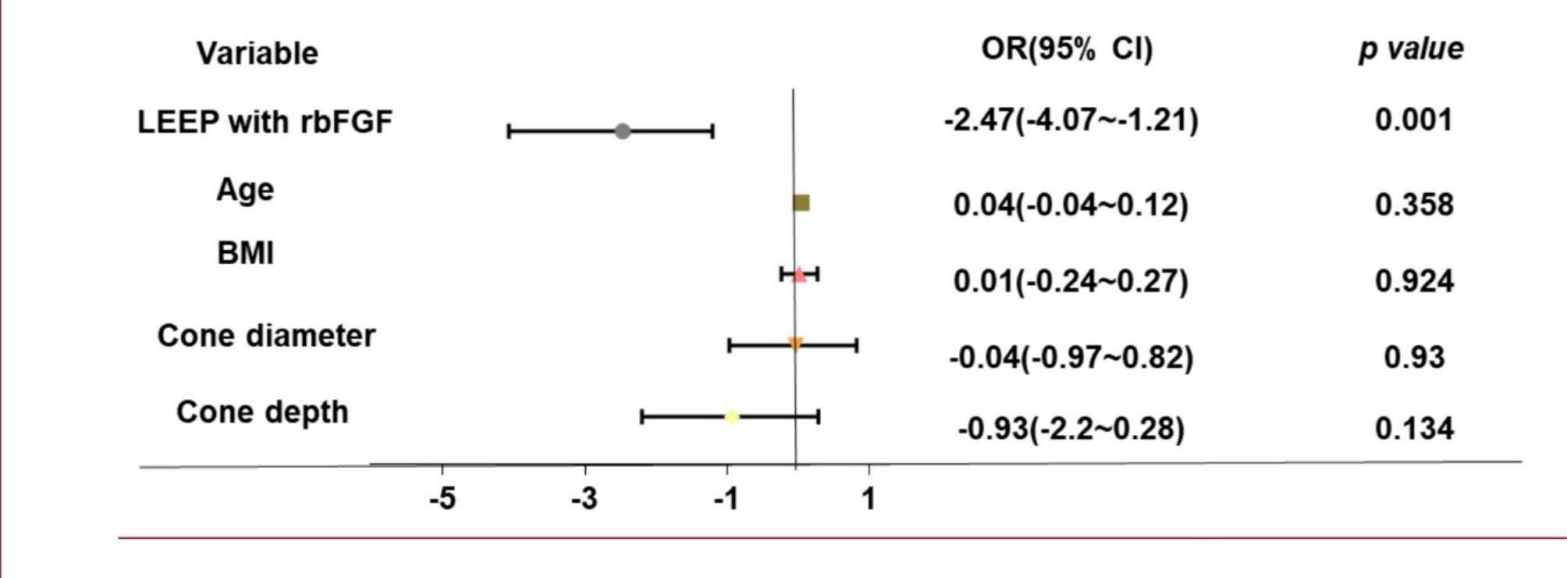
Multivariate analysis: influence of significant variables on postoperative hemorrhage

In the multivariate analysis, with significant bleeding as the dependent variables, the allocation to the study group was found to have a significant influence (*p* < 0.001, OR = -2.47, 95% CI -4.07 ~-1.21) on postoperative hemorrhage, while age, BMI, and surgical specimen did not independently impact the occurrence of postoperative hemorrhage (Fig. 3).

Multiple logistic regression analyses with postoperative hemorrhage, stratified through whether significant bleeding in 6 weeks after the LEEP procedure, as dependent variables and study group (use of rbFGF), age, body mass index, surgical specimen (cone diameter and cone depth) as independent variables.

## Discussion

The use of LEEP is considered the optimal choice compared to other alternatives in terms of recurrence and postoperative infection [1]. However, postoperative hemorrhage remains a significant concern for physicians in outpatient settings. Therefore, reducing the delayed vaginal bleeding after LEEP has become a focus of clinical attention. In this study, we investigated the efficacy of rbFGF in addressing this issue.

While several studies have reported the risk of minor bleeding after LEEP, objective quantification of this bleeding remains lacking. We study the assessment of vaginal blood loss including the alkaline haematin method, the Pictorial Blood Loss Assessment Chart (PBAC), the menstrual diary, and the laboratory data method [13–16]. We propose the use of MVM (measured through menstrual blood loss) as a quantifiable measure for delayed vaginal bleeding. Clinically, we found this method to be easy to implement and the analysis to be objective.

The study by Fu X, et al., published in The Lancet in 1998, revealed the role of rbFGF in reducing healing time and improving healing quality [17]. Subsequently, several clinical studies conducted in China by scientists explored the effects of rbFGF on wound healing [18, 19]. In 2000, the Chinese Food and Drug Administration approved recombinant human basic fibroblast growth factor for the treatment of chronic wounds, including chronic granulating wounds, ulcers, bedsores, traumatic and surgical wounds, and burn wounds, without apparent adverse effects [20].

Our results findings demonstrate that Rb-FGF effectively reduces postoperative bleeding without significant complications. We postulated that rbFGF may exert its effects by promoting wound healing. Wound healing is a complex and dynamic process involving various mechanisms, such as coagulation, inflammation, cellular proliferation, neovascularization, and extracellular matrix remodeling. However, the precise mechanisms underlying the enhanced wound healing and improved healing quality facilitated by exogenous fibroblast growth factors remain unclear [17]. Recent studies have suggested that FGF promotes cell proliferation, reduces inflammatory responses [21, 22], and facilitates angiogenesis [21, 23], among other mechanisms, offering potential insights.

Fibroblast growth factors hold promise in promoting optimal cervical healing postoperatively, thereby reducing delayed vaginal bleeding. Systematic analyses have indicated that surgical treatment of cervical intraepithelial neoplasia (CIN) may have adverse effects on obstetric outcomes, including an increased risk of preterm birth, low birth weight, and preterm premature rupture of membranes before 37 weeks [24]. By improving the quality of cervical healing, the use of rbFGF may positively impact the obstetric outcomes of patients undergoing cervical conization.

Recurrence risk is a critical consideration in cervical conization. Studies have reported that approximately 10.4% of patients with persistent HPV infection and positive surgical margins after conization experienced CIN2 + recurrence during a 5-year follow-up period [25]. Large-scale retrospective multicenter studies have further suggested an increased risk of CIN recurrence in patients with positive high-risk HPV infection [26]. Although the use of rbFGF is generally not believed to prevent recurrence or increase its risk, definitive conclusions require further long-term follow-up.

Our study has several limitations. Firstly, it is a retrospective study, and as such, various biases may have influenced the collected data. Additionally, the small study population is another limitation that should be acknowledged.

In conclusion, rbFGF shows promise as a drug for reducing postoperative hemorrhage after LEEP. Through its potential to promote tissue repair, rbFGF may also mitigate other complications after LEEP such as premature delivery and abortion. Further studies involving a large number of patients and long follow-ups are warranted to validate our findings.

## Data Availability

The datasets generated and/or analyzed during the current study are not publicly available due to the limitations of ethical approval involving the patient data and anonymity but are available from the corresponding author upon reasonable requests.

## References

[1] Santesso N, Mustafa RA, Schünemann HJ, Arbyn M, Blumenthal PD, Cain J (2016). World Health Organization Guidelines for treatment of cervical intraepithelial neoplasia 2–3 and screen-and-treat strategies to prevent cervical cancer. Int J Gynecol Obstet.

[2] Prendiville W, Cullimore J, Norman S (1989). Large loop excision of the transformation zone (LLETZ). A new method of management for women with cervical intraepithelial neoplasia. BJOG Int J Obstet Gynaecol.

[3] Wright TC, Gagnon S, Richart RM, Ferenczy A (1992). Treatment of cervical intraepithelial neoplasia using the loop electrosurgical excision procedure. Obstet Gynecol.

[4] Cohen PA, Leung Y, Anderson L, Van Der Griend R, Chivers P, Bilic S (2020). Excisional treatment comparison for in situ endocervical adenocarcinoma (EXCISE): a phase 2 pilot randomized controlled trial to compare histopathological margin status, specimen size and fragmentation after loop electrosurgical excision procedure and cold knife cone biopsy. Gynecol Oncol.

[5] Santesso N, Mustafa RA, Wiercioch W, Kehar R, Gandhi S, Chen Y (2016). Systematic reviews and meta-analyses of benefits and harms of cryotherapy, LEEP, and cold knife conization to treat cervical intraepithelial neoplasia. Int J Gynecol Obstet.

[6] Hurtado-Roca Y, Becerra-Chauca N, Malca M (2020). Efficacy and safety of cryotherapy, cold cone or thermocoagulation compared to LEEP as a therapy for cervical intraepithelial neoplasia: systematic review. Rev Saúde Pública.

[7] Signorile PG, Anselmi Angeli R (1997). Diagnostic and therapeutic technique of cervical conization with “cold knife” using fibrin glue. Preliminary outcomes. Eur J Gynaecol Oncol.

[8] Martin-Hirsch PP, Kitchener HC, The Cochrane Collaboration (1999). Interventions for preventing blood loss during the treatment of cervical intraepithelial neoplasia. Cochrane database of systematic reviews.

[9] Kim K, Park S-I, Kim B-J, Kim M-H, Choi S-C, Ryu S-Y (2012). Efficacy of Fibrin Sealant in reducing hemorrhage after a Loop Electrosurgical Excision Procedure. Gynecol Obstet Invest.

[10] Kim JH, Park TC, Park GA, Song JY, Kim YH, Lee HJ (2015). A pilot study to investigate the efficacy of Fibrin Sealant (Tisseel®) in the Loop Electrosurgical Excision Procedure. Gynecol Obstet Invest.

[11] Chong GO, Lee YH, Jeon SY, Yang H-Y, An S-H (2020). Efficacy of a chitosan tampon in the loop electrosurgical excision procedure: a prospective randomized controlled study. Sci Rep.

[12] Han C, Cheng B, Wu P, writing group of growth factor guideline on behalf of Chinese Burn Association (2020). Clinical guideline on topical growth factors for skin wounds. Burns Trauma.

[13] Ko JK, Lao TT, Cheung VY (2021). Pictorial blood loss Assessment Chart for evaluating heavy menstrual bleeding in asian women. Hong Kong Med J.

[14] Hald K, Lieng M (2014). Assessment of periodic blood loss: interindividual and intraindividual variations of Pictorial Blood loss Assessment Chart Registrations. J Minim Invasive Gynecol.

[15] Quinn SD, Higham J (2016). Outcome measures for heavy menstrual bleeding. Womens Health.

[16] Schumacher U, Schumacher J, Mellinger U, Gerlinger C, Wienke A, Endrikat J (2012). Estimation of menstrual blood loss volume based on menstrual diary and laboratory data. BMC Womens Health.

[17] Fu X, Shen Z, Chen Y, Xie J, Guo Z, Zhang M (1998). Randomised placebo-controlled trial of use of topical recombinant bovine basic fibroblast growth factor for second-degree burns. The Lancet.

[18] Hui Q, Jin Z, Li X, Liu C, Wang X (2018). FGF family: from Drug Development to Clinical Application. Int J Mol Sci.

[19] Farooq M, Khan AW, Kim MS, Choi S (2021). The role of fibroblast growth factor (FGF) signaling in tissue repair and regeneration. Cells.

[20] Chen K, Rao Z, Dong S, Chen Y, Wang X, Luo Y (2022). Roles of the fibroblast growth factor signal transduction system in tissue injury repair. Burns Trauma.

[21] Álvarez Z, Kolberg-Edelbrock AN, Sasselli IR, Ortega JA, Qiu R, Syrgiannis Z (2021). Bioactive scaffolds with enhanced supramolecular motion promote recovery from spinal cord injury. Science.

[22] Wu F, Wang P, Wei X, Yang Y, Al Mamun A, Zhang X (2023). Barrier-penetrating liposome targeted delivery of basic fibroblast growth factor for spinal cord injury repair. Mater Today Bio.

[23] Yu P, Wilhelm K, Dubrac A, Tung JK, Alves TC, Fang JS (2017). FGF-dependent metabolic control of vascular development. Nature.

[24] Monti M, D’Aniello D, Scopelliti A, Tibaldi V, Santangelo G, Colagiovanni V (2021). Relationship between cervical excisional treatment for cervical intraepithelial neoplasia and obstetrical outcome. Minerva Obstet Gynecol.

[25] Giannini A, Di Donato V, Sopracordevole F, Ciavattini A, Ghelardi A, Vizza E (2023). Outcomes of High-Grade cervical dysplasia with positive margins and HPV persistence after cervical conization. Vaccines.

[26] Bogani G, Sopracordevole F, Di Donato V, Ciavattini A, Ghelardi A, Lopez S (2021). High-risk HPV-positive and -negative high-grade cervical dysplasia: analysis of 5-year outcomes. Gynecol Oncol.

